# Clinical Results and Aortic Remodeling After Endovascular Treatment for Complicated Type B Aortic Dissection With the “Fabulous” Stent System

**DOI:** 10.3389/fcvm.2022.817675

**Published:** 2022-02-14

**Authors:** Ruihan Wang, Yuanqing Kan, Mou Yang, Hongkun Zhang, Xiaoming Zhang, Xiangchen Dai, Shuiting Zhai, Hejie Hu, Xiwei Zhang, Bing Chen, Jianhua Huang, Xiao Qin, Zhanxiang Xiao, Xinwu Lu, Wei Guo, Yi Si, Weiguo Fu

**Affiliations:** ^1^Department of Vascular Surgery, Zhongshan Hospital, Fudan University, Shanghai, China; ^2^Vascular Surgery, Yantai Yuhuangding Hospital Affiliated With Qingdao University, Yantai, China; ^3^Department of Vascular Surgery, The First Affiliated Hospital, School of Medicine, Zhejiang University, Hangzhou, China; ^4^Department of Vascular Surgery, Peking University People's Hospital, Beijing, China; ^5^Department of General Surgery, Tianjin Medical University General Hospital, Tianjin, China; ^6^Department of Vascular and Endovascular Surgery, Henan Provincial People's Hospital, Zhengzhou University, Zhengzhou, China; ^7^Department of Vascular Surgery, The First Affiliated Hospital of USTC, Hefei, China; ^8^Department of Vascular Surgery, Jiangsu Province Hospital, Nanjing, China; ^9^Department of Vascular Surgery, School of Medicine, Second Affiliated Hospital, Zhejiang University, Hangzhou, China; ^10^Department of General and Vascular Surgery, Xiangya Hospital, Central South University, Changsha, China; ^11^Department of Vascular Surgery, The First Affiliated Hospital of Guangxi Medical University, Guangxi, China; ^12^Department of General Surgery, Hainan Province People's Hospital, Haikou, China; ^13^Department of Vascular Surgery, Shanghai Ninth People's Hospital, Shanghai, China; ^14^Department of Vascular and Endovascular Surgery, Chinese PLA General Hospital, Beijing, China

**Keywords:** aortic dissection, aortic remodeling, composite device, thoracic endovascular aortic repair (TEVAR), bare stent

## Abstract

**Objective:**

To report the clinical outcomes and aortic remodeling after the implantation of a self-developed, biomechanically optimized, two-stage thoracic stent system named Fabulous.

**Background:**

Given the efficacy of the PETTICOAT concept, the benefits of Fabulous and the behavior of remodeling in different segments need further investigation.

**Methods:**

This is a prospective and multicenter study. From 2017 to 2019, 145 patients (mean age, 56.6 years; 88.3% male) from 14 centers were included in this cohort. The clinical results and core laboratory results were from a central electronic data capture system. Computed tomographic angiography was performed preoperatively, 1 month, 6 months and yearly thereafter and was used for volumetric analysis by 3mensio (Bilthoven, The Netherlands). After the 1-year follow-up, 97.2 and 87.6% of the clinical and imaging results of the eligible patients were available.

**Results:**

Both stent grafts and bare stents were successfully delivered in place in 100% of the patients. The 30-day mortality and 1-year freedom from all-cause mortality were 2.1 and 96.6%, respectively. The incidence of entry flow was 11.7% at 30 days and 6.2% at 365 days. No cases of stent-induced new entry (SINE) or reintervention were observed. After the 1-year follow-up, the true lumen/overall volume ratio reached 88%. The following subdivided segment volume changes were recorded: stent graft segment TL +56%; FL −92%, bare stent segment TL +32%; FL −75%, and there were no significant changes in the visceral segment.

**Conclusions:**

These outcomes indicated that there were favorable clinical benefits of Fabulous stent system. This device achieved a low short-term mortality and a low incidence of reintervention. In addition, patients undergoing Fabulous stent system implantation showed remodeling both on descending aorta and on the distal aorta. The volume changes in the TL and FL varied in the different segments. The long-term follow-up is still ongoing.

## Introduction

Thoracic endovascular aortic repair (TEVAR) has gradually become the accepted first-line treatment for complicated type B aortic dissection (cTBAD) over the last decades because of its clinical benefits (1–3), and the primary aim of TEVAR is to cover the proximal entry tear, isolate the false lumen (FL) and enhance aortic remodeling. However, in the mid- to long-term follow-up, it was found that the true lumen (TL) expansion and false lumen (FL) thrombosis in some patients were usually limited to the segment covered by the stent graft (SG) (4–6).

An extensive coverage length is preferable, but having an extensive coverage could increase the incidence of paraplegia and visceral ischemia. Therefore, traditional TEVAR still has certain limitations in promoting the aortic remodeling distal to the SG, which could be affected by a persistent distal entry-flow (7–9). When exploring a solution to the contradiction, researchers have tried to combine the traditional proximal SG with a distal bare metal stent together for cTBAD, which is known as the PETTICOAT technique (5, 10, 11). The use of the proximal SG as in traditional TEVAR could seal the primary entry tear, and the distal bare metal stent provides an extensive expansion of the distal TL and ensures the perfusion of critical organs. The composite devices used in existing studies were all Zenith Dissection Endovascular Systems (William Cook Europe, Bjaerverskov, Denmark), which are only available in two sizes and a one-taper design (12). However, the morphology of the aorta and the dissection at the distal end show different conical shapes. Therefore, the diameters of the aorta and the dissection vary among patients, so there are less choices in the two-size and one-taper design for different aortic morphologies. Therefore, our center self-developed a new two-stage stent system named the Fabulous stent system. This system adopted a multi-taper design, and provided a variety of sizes to choose from according to the patient's anatomical characteristics. Furthermore, the design was optimized from the perspective of biomechanics aiming to improve its compatibility. We conducted this multi-center, prospective, single-arm study to verify the safety and efficacy of this system and used volume measurements to demonstrate the effect of the composite stent on the remodeling of the different segments of the aorta.

## Methods

This study was approved by the relevant ethics committees at each center, and the enrolled patients were provided inform consent and medical insurance.

### Study Design and Eligibility

This study was a multi-center, prospective, single-arm study conducted at 14 institutions (unique protocol ID: WQ1601), and this study aimed to evaluate the safety and efficacy of the Fabulous stent system for patients with cTBAD. The endpoint was the 1-year all-cause mortality; the other outcomes included aortic remodeling, device-related complications, clinical utility and device performance. In addition, we used semiautomated segmentation to subdivide the aorta, and measured the volume changes of the TL and FL in different segments to demonstrate the process of remodeling in detail. An interactive web response system (IWRS) was adopted to assign the patient registration numbers. All patients who met the inclusion criteria and patients were excluded if they met any of the exclusion criteria. The detailed criteria were shown in Table 1. All patients received implantation of the Fabulous stent system within 3 months of onset of dissection symptoms.

**Table 1:** Inclusion & exclusion criteria.

**Inclusion**	**Exclusion**
Age >18 years old	Age <18 years
The arterial access was suitable for surgical treatment	Pregnant or breastfeeding
Clinical diagnosis of cTBAD	Participating in another clinical device or drug study
a) The presence of rapid aortic expansion	Inability to sign the informed consent
b) Aortic rupture and/or hypotension/shock	Unwilling to comply the follow-up plan
c) Refractory hypertension despite adequate medication	Diagnosed with congenital connective tissue diseases (Marfan or Ehlers-Danlos syndrome)
d) Paraplegia/paraparesis	Patients with an aortic aneurysm or a pseudoaneurysm
e) Recurrent or refractory pain	Allergy to contrast agents, anesthetics, stent-graft or delivery system
f) Visceral, renal, or limb ischemia	Distal false lumen was completely thrombotic or organized
Proximal landing zone length measuring ≥15 mm	History of aortic surgery or a placement of an endovascular stent-graft
Proximal landing zone diameter for stent graft 18–42 mm	History of MI or TIA within 3 months
	Limited life expectancy (less than 12 months)
	Dissection involving the branches of aortic arch
	Proximal landing zone length measuring <15 mm
	Proximal landing zone diameter for stent graft <18 mm or >42 mm

*cTBAD, complicated type B aortic dissection; MI, myocardial infarction; TIA, transient ischemia attack*.

### Device Description of The Fabulous Stent System and Implantation Procedure

**Figure 1:**
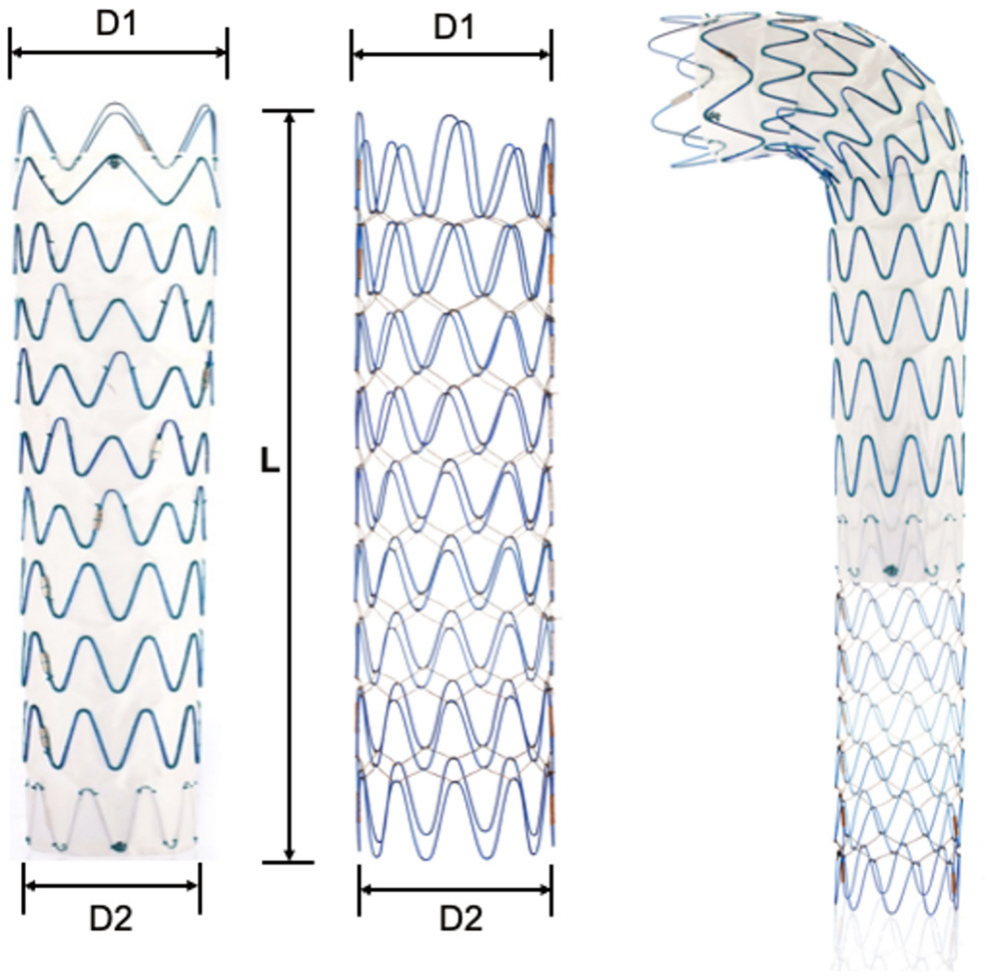
Illustration of the Fabulous system. Fabulous consists of the proximal stent graft and the distal bare stent. D1, the proximal diameter. D2, the distal diameter. L, the length of the stent. Both components are available in 13 sizes (D1) and in tapered and non-tapered configurations (D1-D2).

The Fabulous stent system consisted of two parts: the proximal covered stent graft system and the distal bare stent system. The two-stage design took support force and flexibility into account, which aimed to promote aortic remodeling (Figure 1). The proximal covered stent-graft consisted of Z-shaped nitinol segments and was covered by a polyethylene terephthalate (PET) graft, and the two were stitched together by polyester sutures. The distal bare stent component was made of nitinol segments. Both the SG and bare stents adopted a self-expanding mode after the withdrawal of the delivery sheath. In addition, the SG and bare stent were both designed to have 13 models according to the proximal diameter (the SG ranging from 20 to 44 mm; the bare stent ranging from 16 to 40 mm). In addition, each model was designed to have different distal diameters so that the stent appeared as a straight cylinder or a cone with different tapers. This design made Fabulous suitable for more patients with different anatomical variations. Standard endovascular protocol was used for the implantation of the proximal stent graft, which is basically the same as traditional TEVAR. Generally, an oversizing rate of 0–10% of the SG based on the proximal landing zone was recommended. And the distal SG diameter is based on the average diameter of the aorta (perimeter of the aorta/π). After the proximal stent graft was in place, the bare stent would be used if the patients: (1) had signs of compromised branch vessels; (2) the systolic pressure of the aortic root is 20 mmHg higher than the distal obstructed vessel; (3) FL flow through the secondary tears. The bare stent was delivered and was overlapped by 3–4 cm with the distal part of the SG and the bare stent should land proximally to the origin of the visceral vessels. The proximal diameter of the bare stent was usually 2 mm larger or was the same as the distal diameter of the SG. The selection of the bare stent was ultimately decided based on the physician's clinical judgment.

Compared with the bare stent of the Zenith system, we improved the Fabulous by using the following design aspects (Table 2). First, Fabulous has more sizes to choose from, and it was designed to have 4 different tapers to suit patients with different aortic morphologies (Supplementary Tables S1, S2). Besides, Fabulous has a smaller length of wave circle diameter, making it more flexible. Additionally, the multifilament design ensures that the stent has a stable axial support force.

**Table 2:** Design differences Fabulous vs. Zenith.

**Design**	**Fabulous**	**Zenith**
Proximal diameter	16–40 mm	36, 46 mm
Taper	0–8 mm	8 mm
Rate of oversize	15–30%	20–80%, 21–48%
Length of wave circle	10 mm	20 mm
Sutures	Multifilament	Monofilament

### Patient Follow-Up

The immediate delivery and surgical success rates were assessed intraoperatively. Imaging evidence, physical examinations and core laboratory examinations were obtained before discharge and at 30 days, 6 months, 12 months and yearly thereafter. The computed tomographic angiography (CTA) results, outpatient records and telephone follow-up results were combined to assess the safety and efficacy of the Fabulous stent system.

### TL and FL Assessment

We first evaluated the patients' FL thrombosis and entry flows based on the CTA results according to the standard of previous researches (6, 13, 14). At the same time, the maximum diameters of the patients' thoracic and abdominal aorta were measured. 3mensio Vascular software (Bilthoven, The Netherlands) was used to integrate and reconstruct the CTA results, and we further subdivided the descending aorta into four segments: Segment 1 was defined as the opening of left subclavian artery (LSA) to the distal end of SG; Segment 2 was defined as the distal end of the SG to the opening of the celiac trunk and contained the segment covered by bare stent; Segment 3 was defined as the opening of celiac trunk to the lower renal artery; Segment 4 was defined as the lower renal artery to the bifurcation. We measured the volumes of the TL and FL of these segments by a semi-automated centerline algorithm separately.

### Statistical Analysis

The imaging data, physical examinations and core laboratory examinations were derived from the central database. Statistical analysis was performed by SPSS Ver 21 software. Continuous variables were expressed as the mean ± standard deviation, and categorical variables were expressed by the frequency and percentage. We required a *P* value below 0.05 to claim strong evidence of a difference.

## Results

From December 2017 to October 2019, a total of 145 patients from 14 centers were enrolled in this study. By the time of the 1-year follow-up endpoint, 97.2% (141 of 145) of patients were available for clinical assessment. A total of 87.6% (127 of 144) of patients were available for CT angiography (CTA) imaging measurements.

### Patient Demographics

The average age of the patients was 56.6 ± 12.5 years old (range, 21–85 years old), and the majority of patients were male (88.3%, 128/145). Typical cardiovascular risk factors could be observed in most participants, and 86.2% (125/145) of the patients had hypertension and 51.1% (74/145) of the patients had a smoking history. Table 3 provides the details of patient demographics and comorbidities.

**Table 3:** The patients' demographics and comorbidities.

**Variables**	***N* = 145**
**Demographics**	
Male, *n* (%)	128 (88.3)
Female, *n* (%)	17 (11.7)
Height, mean ± SD (range) cm	169.4 ± 6.8 (150–188)
Weight, mean ± SD (range) kg	73.4 ± 13.9 (40–120)
Age, mean ± SD (range) years	56.6 ± 12.5 (21–85)
**Comorbidities**, ***n*** **(%)**	
CAD	5 (3.4)
LEVF <20%	0 (0.0)
Hypertension	125 (86.2)
Hyperlipidemia	10 (6.9)
Previous endovascular surgery	0 (0.0)
**Smoking**	
Currently smoke	71 (49.0)
Never smoked	71 (49.0)
Quit smoking	3 (2.1)
Length of hospital stay, median (Q1, Q3) days	14 (10, 19)

*CAD, coronary artery disease; LEVF, left ventricular ejection fraction*.

### Dissection Characteristics

The anatomic characteristics of the dissections are shown in Table 4. Primary tears (95.9%, 139/145) in the descending aorta were most prevalent. There were 26.9% (39 of 145) of the patients that had dissections extending to the iliac arteries. The initial largest diameter of the false lumen (FL) was 25.5 ± 12.3 mm (range, 0–71 mm), and the minimum diameter of the true lumen (TL) was 13.9 ± 8.0 mm (range, 0–32 mm).

**Table 4:** Dissection characteristics.

**Variables**	***N* = 145**
Type of dissection, *n* (%)	
Stanford B	145 (100.0)
Location of entry tears*, n* (%)	
Descending aorta	
Primary tears	139 (95.9)
Secondary tears	2 (1.4)
None	4 (2.8)
Above celiac trunk	
Primary tears	9 (6.2)
Secondary tears	64 (44.1)
None	72 (49.7)
Renal-to-iliac artery	
Primary tears	5 (3.4)
Secondary tears	43 (29.7)
None	97 (66.9)
Iliac artery	
Primary tears	5 (3.4)
Secondary tears	43 (29.7)
None	97 (66.9)
Anatomic features	
Proximal sealing zone, mean ± SD (range) mm	
Diameter	29.5 ± 3.4 (20–40)
Length	24.7 ± 15.5 (15–150)
Minimum diameter of TL, mean ± SD (range) mm	13.9 ± 8.0 (0–32)
Maximum diameter of FL, mean ± SD (range) mm	25.5 ± 12.3 (0–71)

*TL, true lumen; FL, false lumen*.

### Mortality

A total of five deaths occurred during the one-year follow-up. The 30-day mortality rate was 2.1% (3 of 145). One patient, who was enrolled with severe chest pain and compromise of his renal arteries, died from upper gastrointestinal hemorrhage 28 days postoperation. This patient had a history of gastric ulcers before enrollment, and a postprocedure DSA showed that the stents were delivered in place without entry flow. The patient's medical history on the day of death showed that he was admitted to the emergency department due to severe hematemesis and melena, and this death was not related to surgery or stent implantation. The second patient, who was enrolled with severe chest pain and compromise of his visceral and renal arteries, died 10 days post-operation during the same hospitalization. He presented to the emergency department unconscious accompanied by profuse sweating. An emergency CTA indicated a type A aortic dissection, but the patient's family decided to abandon the rescue and the patient died at discharge. This patient's death may be related to surgery and stent implantation. The third patient, who was enrolled with chest tightness, pleural effusion and lower limb weakness, died 1 day postoperation. This patient had chest pain, was unconscious and was hypoxemic. The patient's family decided to abandon treatment and elected not to have an autopsy performed, and this patient died of unknown reasons. According to this patient's medical history, the clinical committee judged that the death may be related surgery and stent implantation.

The 1-year freedom from all-cause mortality was 96.6%. Two additional patients died between 30 and 365 days. One patient was enrolled with persistent chest pain and impaired renal function and did not respond well to medical management. He was admitted at 204 days after surgery and presented with abdominal pain and vomiting. An emergency CTA showed the stents were in place and that the false lumen was completely thrombotic, but he also had a dilated bowel that indicated an intestinal obstruction. Subsequently, the patient died 208 days post-operation. And this death is not related to stent implantation but may be related surgery. The other patient was enrolled with severe abdominal pain and a transaortic diameter of 54 mm and died 148 days post-operation of unknown reasons. This patient went to our center for a CTA examination at 30 days, and he recovered well without any complaints of discomfort. However, through the telephone follow-up, it was noted that the patient had died at home without an autopsy performed. Because of the unknown cause of death, this death was unable to be adjudicated. Table 5 summarizes the 5 deaths and the clinical events committee adjudications.

**Table 5:** Overview of the patient deaths.

**Patients' number**	**Time (days)**	**Cause of death**	**Related to surgery**	**Related to stent graft**
29	28	Gastrointestinal hemorrhage	Not related	Not related
73	10	TAAD	Maybe related	Maybe related
99	1	Unknown	Maybe related	Maybe related
85	148	Unknown	Unable to be adjudicated	Unable to be adjudicated
132	208	Intestinal obstruction	Maybe related	Not related

*TAAD, type A aortic dissection*.

### Entry Flow, Stent Migration, Stent Integrity, Stent-Induced New Entry (SINE) and Reintervention

A total of 11 type I entry flows (7.6%) and 6 type II entry flows (4.1%) were observed 30 days after the initial procedure. During the 1-year follow-up period, 8 type I entry flows and 4 type II entry flows decreased significantly at 180 and 365 days. There were 7 type I entry flows (4.8%) at 365 days (4 of them were newly developed) and 2 type II entry flows (1.4%). Compared with the 30-day CTA, the clinicians considered that these entry flows were minor, and the diameters of TL and FL had not changed significantly. Therefore, there was no reintervention for these entry flows. There were no cases of stent migration, stent fracture, stent rupture or in-stent thrombosis. Based on the 1-year CTAs, no cases of SINE were observed. In addition, because no vital organ ischemia or increased FLs due to entry flows were observed. The incidence of at least one re-intervention after surgery was 0%. The follow-up work on intreated entry flows is still ongoing.

### Aortic Remodeling

Based on the 1-year CTA assessment, patients undergoing the Fabulous system implantation showed a favorable extent of false lumen thrombosis. The false lumen thrombosis was absent in all patients preoperatively. A total of 67.6% of patients (98 of 141) had partial false lumen thrombosis at 30 days. A total of 41.1% of patients (60 of 138) had partial false lumen thrombosis at 1 year after endovascular surgery. However, the rate of complete false lumen thrombosis increased from 29.7% (43 of 141) at 30 days to 53.8% (78 of 138) at one year (Table 6).

**Table 6:** False lumen status.

**Status**	**Preoperative % (n/N)**	**30 days % (n/N)**	**6 months % (n/N)**	**1 year % (n/N)**
No thrombosis	100 (145/145)	0 (0/141)	0 (0/131)	0 (0/138)
Partial thrombosis	0 (0/145)	67.6 (98/141)	46.2 (67/131)	41.1 (60/138)
Complete thrombosis	0 (0/145)	29.7 (43/141)	44.1 (64/131)	53.8 (78/138)

All participants demonstrated a significant increase in the TL diameter and a decrease in the FL diameter when using the largest diameter segment in both the thoracic and abdominal aorta. There was a statistically significant difference in the changes in the TL diameter (+10.06 mm in the thoracic aorta and +7.57 mm in the abdominal aorta; *P* < 0.01), as well as in the FL diameter (−11.58 mm in the thoracic aorta and −6.74 mm in the abdominal aorta; *P* < 0.01).

The patients showed varying degrees of positive remodeling in terms of the TL/overall volume ratio when compared to the preoperative status. In the early-term postoperative period, all patients began to demonstrate an increase in the TL volume and a decrease in the FL volume. The total TL/overall ratio increased from 72% preoperatively to 84% one month after surgery (*P* < 0.05). At the 6-month follow-up, the changes in the TL and FL volumes reached a relatively stable level. Compared with the 1-year follow-up, the TL/overall ratio did not show a significant change (6-month: 88 ± 12%; 1-year: 88 ± 9%).

**Figure 2:**
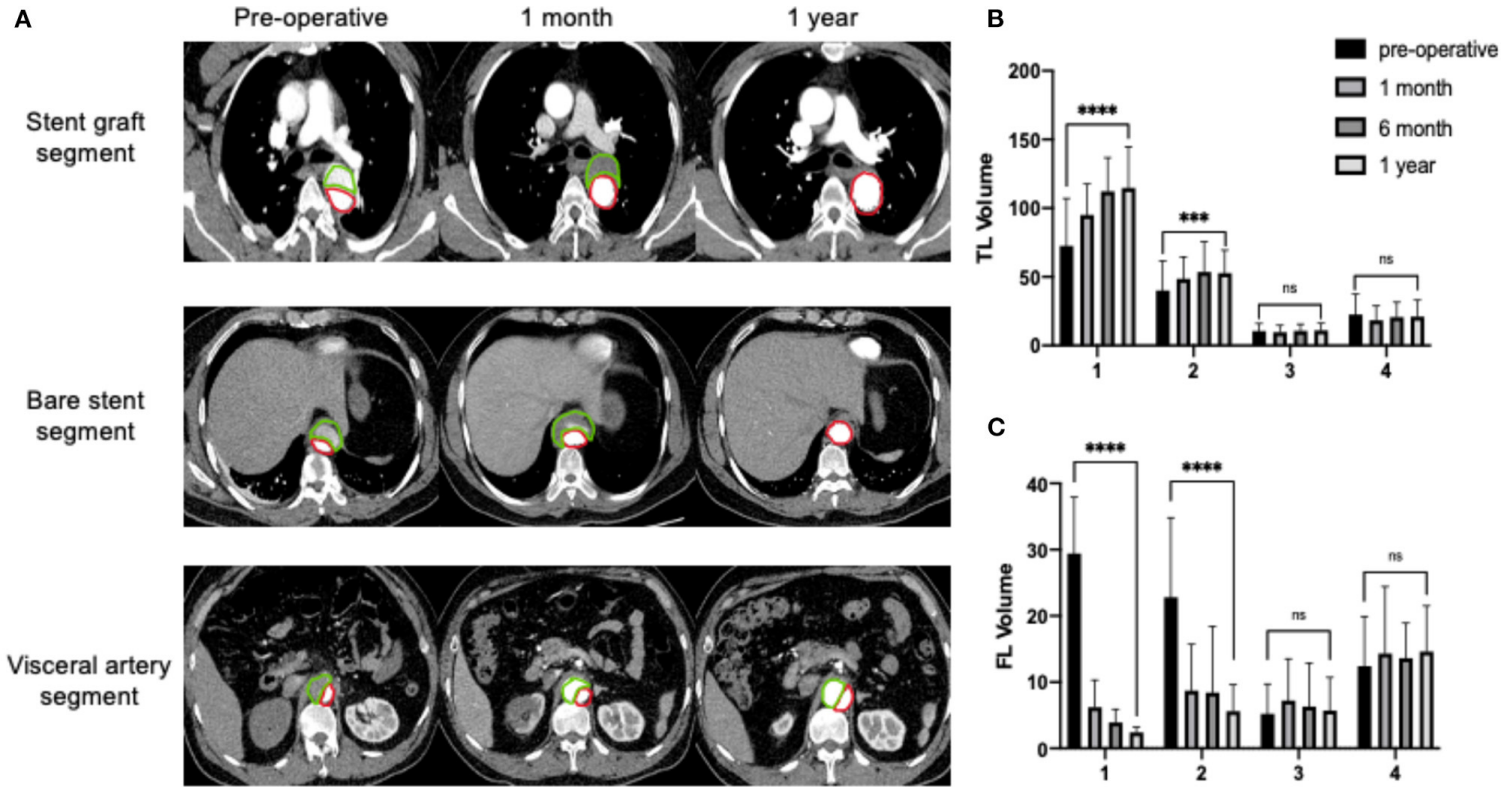
Continuous process of aortic remodeling with an expanding TL and shrinking FL after the implantation of the Fabulous stent system. Segment 1: the opening of LSA to the distal end of SG; Segment 2: the distal end of the SG to the opening of the celiac trunk and containing the segment covered by bare stent; Segment 3: the opening of celiac trunk to the lower renal artery; Segment 4: the lower renal artery to the bifurcation. **(A)** The true lumen (TL, red) gradually expanded and the false lumen (FL, green) gradually shrank. **(B)** The volume of the TL (cm3) continuously increased in segments covered by stent grafts and bare stents, and reached a relative high level at 6th month and reached a stable level at 1 year. The uncovered segments didn't show the trend of negative remodeling. **(C)** The volume of FL (cm3) decreased significantly in segments covered by stent graft and bare stent. No increase in volume of the uncovered FL. ***P < 0.001; ****P < 0.0001.

According to the further assessment of the volume changes and the analysis of subsegments, different behaviors in the volume changes were observed. One year after the procedure, the TL volume of the segment covered by SG (segment 1) increased by 56% (^****^P < 0.0001), the segment covered by bare stent (segment 2) increased by 32% (^***^P < 0.001) and the TL volume increased by 5% (*P* > 0.05) and decreased by 6% (*P* > 0.05) in segments 3 and 4, respectively. The volume of the FL covered by SG (segment 1) decreased by 92% (^****^P < 0.0001) and the segment covered by bare stent (segment 2) decreased by 75% (^****^P < 0.0001), but the volume of the FL increased by 9% (*P* > 0.05) and 7% (*P* > 0.05) in segments 3 and 4, respectively (Figure 2).

## Discussion

We conducted this multi-center, prospective study using our self-designed Fabulous system for the endovascular repair of cTBAD patients. From our results, we found that using the two-stage design of the Fabulous system derived from the PETTICOAT concept in cTBAD patients can achieve remarkable aortic remodeling with TL expansion and FL shrinkage, which were comparable with the related studies to date. In particular, we used volume measurements to evaluate the changes in the TL and FL, and provided a more detailed and comprehensive remodeling process. In addition, the optimization of biomechanics of the bare stents may reduce the incidence of reintervention.

The ideal goal of TEVAR is to seal the primary tear and to induce FL thrombosis and shrinkage. However, to prevent serious complications, such as paraplegia caused by spinal ischemia, the coverage length is usually limited with the distal tears putting-aside. As a result, sometimes positive remodeling of the distal segment is not ideal since traditional TEVAR cannot address the ongoing perfusion of distal entry, which may be an incipient risk leading to long-term reintervention or even death after surgery. The method of combining the distal bare mental stent helped to solve this dilemma to some extent.

From the perspective of safety, previous clinical trials on the PETTICOAT technique reported a low average 30-day mortality of 4.9% (ranging from 0 to 17.6%), and the survival rates 1 year after the procedure ranged from 80 to 93.3% (15–17). Our 2.1% 30-day mortality rate and 96.6% rate of freedom from all-cause death at 1 year were comparable with these similarly structured studies. In addition, no patients developed paraplegia, kidney failure, bowel ischemia, myocardial infarction or infection of stent throughout the 1-year follow up in our cohort.

Of all the participants, no cases of reintervention were reported, which was lower than the results of STABLE II and the results from Lombardi et al.'s study, with the incidences of 12.3 and 17.1%, respectively (15–18). An early reintervention was mainly related to type I entry flow in their studies, which was also observed in our study. Differently, the Fabulous stent system fit well with the aortic morphologies of different patients because of it has a multi-taper design and has a variety of bare stents to choose from. Most of the entry flows were minor ones, and we did not pursue aggressive treatments for them. We used a close follow-up protocol for the observed entry flows, and these entry flows did not show any further enlargement. Furthermore, among their reported reinterventions, 10 SINEs required reintervention, which was not observed in our study. SINE is a noteworthy device-related complication that usually requires reintervention (19, 20). Optimizing the relationship between the elastic stress of the stent and the shape of the aortic dissection is receiving increasing attention to reduce the incidence of SINE. The lower reintervention rate of the Fabulous system may also be related to its different design compared with Zenith. From a biomechanical point of view, Fabulous has a more stable radial support force and smaller elastic recoil force, which are believed to play a major role in the occurrence of SINE (19, 21, 22). First, Fabulous adopted a multi-taper design with a 15–30% rate of oversizing. The Zenith stent had only one taper and the oversizing rate were 20–80% for 36 mm stent and 21–48% for 46 mm stent, respectively. It can be inferred that the multi-taper design was more in line with the conical shape of the aorta and that the oversize range of Zenith was relatively large, which led to the an uneven radial support force on the dissections of different diameters (20, 23). Second, Fabulous has a smaller length of wave circle, and its multifilament suture makes the stent more compliant. Therefore, Fabulous possesses a lower elastic recoil force, and the damage to the intima was reduced. Moreover, there was study used computational hemodynamics and 3D structural analysis to illustrate the flow and the wall motion (24). And they reported that the first balance position of the lumen pressure played a critical role in aortic remodeling, which is a novel point of view. This method could be applied in future study to investigate whether there are differences between Fabulous system and traditional TEVAR in influencing the magnitude of shifting of the balance position. The above biomechanical optimizations may partially explain the lower rate of reinterventions after using the Fabulous stent system, but the specific biomechanics and long-term efficacy of this device remain to be verified by further studies.

A search of the literature revealed scant studies evaluating aortic remodeling by assessing the volume changes. The commonly used parameters included the FL thrombosis (patent, partial or complete), the largest diameter of TL or FL and the area of TL or FL of a certain segment, which all had their own defects. The FL thrombosis is the most common qualitative indicator, but it can only provide subjective and discontinuous results. The most commonly used quantitative parameter is the TL/FL diameter, but when the aorta is remodeling, the location of the maximum diameter of the TL or FL may change. Therefore, the comparability of the different periods is not strong. In addition, the shape of the dissection is usually irregular, so measuring the diameter may not replicate of the exact condition of TL or FL. Furthermore, the thoracic aorta is a conical shape, and the SG is also designed as a conical structure. However, the diameter measurement could only represent a certain cross section, and the maximum diameter section of TL and FL varies from patients. Therefore, there is a lack of horizontal comparability. Combining the above limitations, the interpretations of the common methods are one-sided and are not sensitive enough. Volume measurement therefore has been affirmed to be a superior choice and could provide the whole picture of remodeling with continuous variable results.

Current studies on the volume assessment after the implantation of a composite device design stated the following common conclusion. First, an immediate increase in TL and a shrinkage of the FL could be observed after the composite stent implantation. This would be a continuous process lasting for more than at least 2 years (4). On average, the overall volume of TL could be increased by more than 100% at one year and could cause a significant decrease in FL likewise. The remodeling of the thoracic aorta was generally more obvious, and the changes were greater than those of the abdominal aorta (25). Our volume assessment of the aorta after the implantation of Fabulous got consistent results that were mentioned above.

Innovatively, we further subdivided the aorta into four parts according to the stent-covered segment and the main branch vessels because the distal end greatly affects the prognosis. In previous studies, the segments of aorta were only roughly divided into two parts: the thoracic aorta and abdominal aorta, so the SG segment, bare stent segment and visceral artery segments were not analyzed in detail. According to our outcomes, adding a bare stent had a greater impact on the thoracic aorta distal to the SG than on the visceral artery segment. However, whether the addition of a bare stent has a more positive aortic remodeling effect in the visceral artery segment than implanting SG alone still needs to be confirmed by further controlled trials.

Our outcomes further confirmed that the composite device could induce an ideal remodeling of the thoracic aorta and that the visceral artery segment did not show negative remodeling. The volume changes basically reached a high level at 6 months and reached a stable level at 1 year. The percentage of the TL to the total aorta could reach 88% at 6 months. The 1-year follow-up reported a continuous positive remodeling, showing that the volumes of TL and FL were further improved. Additionally, we found that the positive remodeling was most obvious at the segment covered by the proximal stent graft (TL: +56%; FL: −92%, at 1 year), followed by the segment covered by the bare stent segment (TL: +32%; FL: −75%, at 1 year). The volume from the visceral segment to bifurcation didn't change significantly, but it remained stable during the 1-year follow-up, and did not show a trend of negative remodeling. Compared with traditional TEVAR, the extended coverage of bare stents can improve the perfusion at the distal end of SG and can promote the expansion of the TL, and patients could benefit more than use SG only (25).

### Limitations

This is a single-arm study, and there is still a need for further comparative studies to verify the current conclusions and to explore the potential advantages of the PETTICOAT technique. In addition, because Zenith's bare stent has not yet been listed on the Chinese market, our results can only be compared with previous similar structured studies, and there is still a paucity of horizontal comparisons. In addition, the impact of two-stage stent and traditional TEVAR on the remodeling of different segments of the aorta remains to be explored. In addition, in our future study, we will explore long-term follow-up results and explained the clinical benefits with a larger amount and further categorized patients.

## Conclusions

The safety and effectiveness of Fabulous could bring clinical benefits to patients. Compared with similar products, the stent system has a comparably low 30-day and 1-year mortality rate. According to the volumetric analysis, a different remodeling behavior was observed in different segments. It was found that the proximal stent graft could effectively expand true lumen and shrink false lumen. The distal bare stent provided extensive expansion of the true lumen and reduced the distal flow in the false lumen. This composite device showed remodeling both on descending aorta and on the distal aorta.

## Perspectives

### What Is Known?

Previous studies have indicated that proximal stent grafts with an extensive coverage of the bare stents could solve the dilemma of having a long coverage leading to ischemia of the vital organs but a short coverage leading to persisting distal false lumen flow.

### What Is New?

We demonstrated that patients who had the Fabulous system had a comparably low short-term mortality. After biomechanical optimization, the design with better flexibility and more stable radial support force reduced the incidence of SINE and reintervention. Furthermore, our segmental volumetric analysis found that there are differences in the remodeling behavior of different parts of the aorta, and the distal bare stent could effectively promote the remodeling of the aorta distal to the stent graft and could reduce the ischemia of vital organs.

### What Is Next?

Long-term follow-up work for the patients who had Fabulous is still ongoing. Compared with traditional TEVAR, the hemodynamic changes of the composite stent and the effect of such changes on remodeling and volume changes remain to be studied.

## Data Availability Statement

The original contributions presented in the study are included in the article/Supplementary Material, further inquiries can be directed to the corresponding authors.

## Ethics Statement

The studies involving human participants were reviewed and approved by the Committee for the Protection of Human Subjects at Zhongshan Hospital Fudan University. The patients/participants provided their written informed consent to participate in this study.

## Author Contributions

RW and YK collected data and performed data analysis and article writing. WF and YS designed the device and the research and modified the manuscript. Other authors conducted the device implantation and participated in data collection. All authors contributed to the article and approved the submitted version.

## Funding

This study was funded by the scientific research funding of National Key R&D Project (2020YFC1107702).

## Conflict of Interest

The authors declare that the research was conducted in the absence of any commercial or financial relationships that could be construed as a potential conflict of interest.

## Publisher's Note

All claims expressed in this article are solely those of the authors and do not necessarily represent those of their affiliated organizations, or those of the publisher, the editors and the reviewers. Any product that may be evaluated in this article, or claim that may be made by its manufacturer, is not guaranteed or endorsed by the publisher.

## Abbreviations

TEVAR: thoracic endovascular aortic repair
TBAD: type B aortic dissection
TL: true lumen
FL: false lumen
SG: stent graft
IWRS: interactive web response system
MAE: major adverse events
SINE: stent-induced new entry.
