# Supplementary material for: Clinical Results and Aortic Remodeling After Endovascular Treatment for Complicated Type B Aortic Dissection With the “Fabulous” Stent System
Source: Front Cardiovasc Med. 2022 Feb 14;9:817675. doi: 10.3389/fcvm.2022.817675 (PMC8882966; doi:10.3389/fcvm.2022.817675)
Supplement: Supplementary file 1 [file Data_Sheet_1.docx]

Supplementary Table 1 (Table S1). Specification sheet of proximal stent graft

| Specification | Proximal diameter  (D1) | Distal diameter  (D2) | Length  (L) | Delivery system |
| --- | --- | --- | --- | --- |
| 20 | 20 | 20、16 | 60、80、100、120、140、160 | 20F |
| 22 | 22 | 22、18 |  | 20F |
| 24 | 24 | 24、20 |  | 20F |
| 26 | 26 | 26、22 |  | 20F |
| 28 | 28 | 28、24、22、20 | 60、80、100、120、140、160、180、200、250 | 20F |
| 30 | 30 | 30、26、24、22 |  | 20F |
| 32 | 32 | 32、28、26、24 |  | 20F |
| 34 | 34 | 34、30、28、26 |  | 20F |
| 36 | 36 | 36、32、30、28 |  | 22F |
| 38 | 38 | 38、34、32、30 |  | 22F |
| 40 | 40 | 40、36、34、32 |  | 22F |
| 42 | 42 | 42、38、36、34 |  | 22F |
| 44 | 44 | 44、40、38、36 |  | 22F |

Supplementary Table 2 (Table S2). Specification sheet of distal bare stent

| Specification | Proximal diameter  (D1) | Distal diameter  (D2) | Length  (L) | Delivery system |
| --- | --- | --- | --- | --- |
| 16 | 16 | 16 | 60、80、100、120、140、160、180、200 | 16F |
| 18 | 18 | 18 |  | 16F |
| 20 | 20 | 20、16 |  | 16F |
| 22 | 22 | 22、18 |  | 16F |
| 24 | 24 | 24、20、18 |  | 16F |
| 26 | 26 | 26、22、20 |  | 16F |
| 28 | 28 | 28、24、22、20 |  | 16F |
| 30 | 30 | 30、26、24、22 |  | 16F |
| 32 | 32 | 32、28、26、24 |  | 16F |
| 34 | 34 | 34、30、28、26 |  | 16F |
| 36 | 36 | 36、32、30、28 |  | 16F |
| 38 | 38 | 38、34、32、30 |  | 16F |
| 40 | 40 | 40、36、34、32 |  | 16F |
